# Quantifying the impact of *Wolbachia* releases on dengue infection in Townsville, Australia

**DOI:** 10.1038/s41598-023-42336-2

**Published:** 2023-09-11

**Authors:** Samson T. Ogunlade, Adeshina I. Adekunle, Michael T. Meehan, Emma S. McBryde

**Affiliations:** 1 Australian Institute of Tropical Health and Medicine, James Cook University, Townsville, Australia; 2 College of Medicine and Dentistry, James Cook University, Townsville, Australia; 3 Department of Defence, Defence Science and Technology Group, Melbourne, Australia; 4 College of Public Health, Medical and Veterinary Sciences, James Cook University, Townsville, Australia

**Keywords:** Applied mathematics, Viral infection, Programming language

## Abstract

From October 2014 to February 2019, local authorities in Townsville, North Queensland, Australia continually introduced *Wolbachia*-infected mosquitoes to control seasonal outbreaks of dengue infection. In this study, we develop a mathematical modelling framework to estimate the effectiveness of this intervention as well as the relative dengue transmission rates of *Wolbachia*-infected and wild-type mosquitoes. We find that the transmission rate of *Wolbachia*-infected mosquitoes is reduced approximately by a factor of 20 relative to the uninfected wild-type population. In addition, the Townsville *Wolbachia* release program led to a 65% reduction in predicted dengue incidence during the release period and over 95% reduction in the 24 months that followed. Finally, to investigate the potential impact of other *Wolbachia* release programs, we use our estimates of relative transmissibility to calculate the relationship between the reproductive number of dengue and the proportion of *Wolbachia*-infected mosquitoes in the vector population.

## Introduction

Dengue viral infection, which is transmitted by *Aedes* mosquitoes has received global attention recently due to its rise and reemergence^1–3^. Of all the *Aedes*-borne diseases, dengue has the most widespread geographical distribution with around 4 billion people at risk and approximately 400 million annual infections^1,4,5^. According to estimates, one in every four dengue cases is symptomatic and notifiable^6^. Dengue transmission dynamics are influenced by various factors such as seasonal variations in weather conditions, vector population density and human population mobility patterns^7^. Dengue epidemiological outbreaks are typically caused by the importation of dengue-infected individuals and occur seasonally in locations where the climate is significantly seasonal^8–10^. The importation of dengue cases has led to the development and reemergence of dengue in a number of nations^11,12^. The global target set by leaders and other partners involved in dengue control programmes such as The World Health Organisation (WHO), research and funding agencies, for dengue infection is to reduce morbidity and mortality by a quarter and a half respectively^13^. This has prompted the development of new control strategies such as *Wolbachia*-based control in the fight against dengue and other *Aedes*-borne diseases such as Zika, chikungunya and yellow fever^14–18^.

*Wolbachia,* an intracellular bacterium, which exists in more than half of all insect species, has been shown to successfully suppress the transmission of dengue viruses in blood-feeding arthropods such as mosquitoes^19,20^. There are several strains of *Wolbachia* such as *w*Mel, *w*AlbA, *w*AlbB, *w*Au and *w*MelPop^21–23^. Mosquitoes bearing different strains of *Wolbachia* have been introduced into the wild, but ones hosting the *w*Mel-*Wolbachia* strain are the most often used variety^14,22–25^. While the *w*Mel *Wolbachia* rollout method has demonstrated highly positive results in reducing dengue-carrying vectors^26,27^, it is not without risk due to the problem of its establishment or stability, as *Wolbachia*-infected mosquitoes are unable to transmit *Wolbachia* maternally to their offspring under high temperature conditions^21,28^.

Mosquitoes bearing the *w*Mel strain of *Wolbachia* were released in Townsville, North Queensland, Australia from October 2014 for 28 months^14^. The *Wolbachia*-infected mosquito introductions led to the replacement of wild-type mosquitoes^14,27^. This *Wolbachia*-based strategy was accompanied by a significant reduction in dengue incidence (estimated around 95% [95% CI 84–98%]), despite an increase in the number of reported imported cases^14,27^. Similar success was reported in nearby Cairns, North Queensland where *w*Mel-infected mosquitoes replaced the wild-type *Aedes aegypti* population (the main vector agent for dengue transmission)^19^. Other countries such as Colombia, Indonesia and Vietnam have rolled out different strains of *Wolbachia* in mosquitoes for their large-scale fight against *Aedes*-borne diseases and have recorded high success rates in mitigating dengue burden^29–31^.

Despite the observed success of *Wolbachia* release programs in reducing dengue incidence, some studies have shown that the *w*Mel *Wolbachia*-infected mosquitoes may lose their *Wolbachia* infections as a result of seasonal fluctuations^21,32^, or fail to significantly reduce dengue incidence especially in high dengue endemic settings^33^. A study^17^ conducted a large *w*Mel-*Wolbachia* release program for a 29-month period (from August 2017 to December 2019), across various locations in Rio de Janeiro, Brazil. Each day, approximately 100 *w*Mel *Wolbachia*-infected mosquitoes were released. The study^17^ estimated the impact on the incidence of dengue and chikungunya, finding that *w*Mel reduced dengue incidence by 38% [95% CI 32–44%] and chikungunya incidence by 10% [95% CI 4–16%]. These reductions are considerably smaller than those observed in other studies^14,23,34^ where dengue incidence fell by 80%. Despite numerous releases, it is unknown why the intervention did not significantly reduce the incidence of vector-borne diseases in Rio de Janeiro^17^. Townsville, a city in North Queensland with population of about 187,500 residents, has climatic seasonal fluctuations which may affect mosquito abundance^35^, such as changes in the carrying capacity of the mosquito population. These changes may threaten the sustainability of the *Wolbachia*-based strategies in controlling arboviral infections.

In this study, we analyze the ‘before’ and ‘after’ *Wolbachia* mosquito introductions (i.e., pre- and post-*Wolbachia* respectively) states in Townsville and estimate the impact on dengue incidence. To do so, we model both the human dengue transmission dynamics alongside the mosquito population dynamics in the presence of *Wolbachia* infection. Other models have described the ecological dynamics of the *Wolbachia*-infected mosquito population only^36,37^ and both *Wolbachia* and dengue dynamics in humans concurrently^33,34^. Here, we extend the *Wolbachia*-mosquito models in^36,37^ via incorporating human populations and dengue infection dynamics, and extend models^33,34^ to include the locally-acquired and imported dengue cases’ compartments to quantify the impact of *Wolbachia* releases on dengue infection in Townsville. Our model estimates the dengue transmission probabilities per mosquito bite between humans and non-*Wolbachia*, and *Wolbachia-*infected mosquitoes and in turn provides insight on the impact of *Wolbachia* introduction on dengue incidence.

## Methods

### Data source and description

#### *Wolbachia* rollout

*Wolbachia* rollout data used in this study were obtained via the record of *Wolbachia* deployment in Townsville^14^. This article described the *Wolbachia* field trials in 32 suburbs in the city of Townsville, which is one of the largest cities in North Queensland, Australia with a population of approximately 187,500^35^. From October 2014, *w*Mel-*Wolbachia*-infected mosquitoes were continually released for a 28-month period. Releases were carried out using mosquito release containers—Mozzie boxes and BioGents Sentinel mosquito traps^14^, set up for subsequent mosquito capture^14^. These traps were monitored and collected weekly prior to February 2016, after which a fortnightly collection ensued. In each release location, *Wolbachia* releases were maintained until the frequency of *Wolbachia-*infected mosquitoes remained above 50% for 2 weeks. Further details on the Townsville *Wolbachia* rollout can be found in^14^. The data provided included the 32 suburbs in which the *Wolbachia* release occurred, the release period, the date and total number of trapped mosquitoes caught and the proportion of *Wolbachia*-infected mosquitoes from the total mosquitoes caught. This data was aggregated into monthly counts to capture the proportion of *Wolbachia*-infected mosquitoes in Townsville.

#### Dengue incidence

The Townsville dengue case notifications data (for locally acquired and imported cases) used for this analysis were extracted from O’Neill et al.^14^. Originally, the information regarding all laboratory-confirmed and clinically probable diagnosis of symptomatic dengue from the beginning of the year 2001 to the first quarter of 2019 was supplied by the Communicable Disease Branch of Queensland Health^38^. These data described the dengue case notifications in Townsville by month of illness onset and history of recent foreign travel by individuals in the 3–12 days before illness onset^14^.

Given that the *Wolbachia* rollout began in October 2014, these dengue cases were stratified into “pre-*Wolbachia*” and “post-*Wolbachia*” periods which translated to cases from January 2001 to September 2014 and October 2014 to February 2019, respectively.

#### Mathematical model of mosquito population and dengue transmission dynamics

Here, a mathematical model is presented (Eq. 1), describing the system of differential equations governing the dengue infection dynamics in the human alongside mosquito population dynamics in the presence of *Wolbachia* infection.

**Figure 1:**
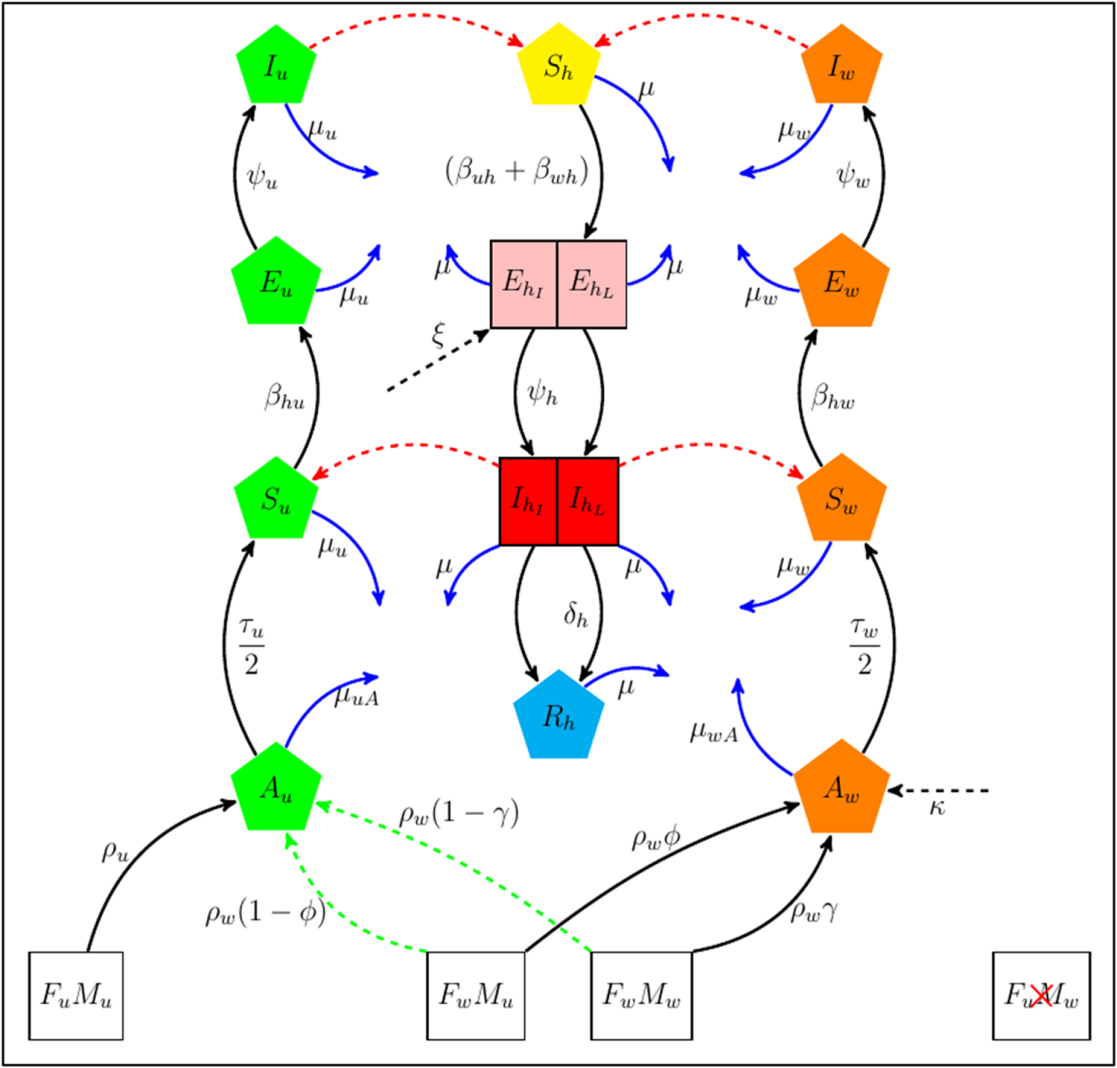
Model formation schematic of dengue infection dynamics between the human population and mosquitoes, which includes the *Wolbachia*-infected mosquitoes. The black solid lines represent the population progression i.e., movement of individuals from one state to another, while the blue solid lines indicate death. In addition, the dashed red lines signify the transmission of dengue infection either from dengue-infected mosquitoes to susceptible humans or vice versa. The dashed green lines are the proportion of uninfected offspring due to imperfect maternal transmission of *Wolbachia* infection. The dashed black lines represent time-varying importations of dengue-infected humans ($\xi$) or *Wolbachia*-infected mosquitoes’ importation ($\kappa$) i.e., the rate at which *Wolbachia*-infected mosquitoes are being released. The $F_{u\left( w \right)}$ and $M_{u\left( w \right)}$ combinations represent the possible mating pairs and generation of offspring from non-*Wolbachia* (*Wolbachia*-infected) mosquitoes respectively. Of these combinations, $F_{u} M_{w}$ does not produce viable offspring due to cytoplasmic incompatibility^39^.

The total human population ($N_{h}$) is divided into subpopulations of number of susceptible individuals ($S_{h}$), individuals exposed to dengue locally ($E_{{h_{L} }}$) and from importation ($E_{{h_{I} }}$), individuals infected with dengue locally ($I_{{h_{L} }}$) and from importation ($I_{{h_{I} }}$), and recovered humans ($R_{h}$). The flow chart representation is illustrated in Fig. 1. Alongside the human dengue infection dynamics, we also model the ecological and infection dynamics of the vector population. To account for the contribution of the number of mosquito vectors and the *Wolbachia* introduction and efficacy, the subpopulation of non-*Wolbachia* mosquitoes is defined as: aquatic juvenile mosquitoes which include larvae and pupae ($A_{u}$), susceptible mosquitoes ($S_{u}$), exposed mosquitoes ($E_{u}$), and dengue-infected mosquitoes ($I_{u}$), while the *Wolbachia*-infected mosquito counterparts are correspondingly subdivided into $A_{w} , S_{w}$, $E_{w}$, and $I_{w}$ (Fig. 1). Further, to make the system simpler, we assume that the ratio of male to female mosquitoes is the same i.e., $M = F$ (resulting in the $\tau /2$ factor in the aquatic maturation flow)^40,41^. Therefore, the adult mosquito state variables for either non-*Wolbachia* $\left( . \right)_{u}$ or *Wolbachia* mosquitoes $\left( . \right)_{w}$ can be written as $F_{u\left( w \right)} = S_{u\left( w \right)} + E_{u\left( w \right)} + I_{u\left( w \right)}$.

The differential system describing the dengue transmission dynamics in humans and mosquito vectors in the presence of *Wolbachia* is given below as.

$$ \frac{{dS_{h} }}{dt} = {\Lambda } - \left( {\beta_{uh} + \beta_{wh} } \right)S_{h} - \mu S_{h} $$

$$ \frac{{dE_{{h_{I} }} }}{dt} = \xi \left( t \right) - \left( {\psi_{h} + \mu } \right)E_{{h_{I} }} $$

$$ \frac{{dE_{{h_{L} }} }}{dt} = \left( {\beta_{uh} + \beta_{wh} } \right)S_{h} - \left( {\psi_{h} + \mu } \right)E_{{h_{L} }} $$

$$ \frac{{dI_{{h_{I} }} }}{dt} = \psi_{h} E_{{h_{I} }} - \left( {\delta_{h} + \mu } \right)I_{{h_{I} }} $$

$$ \frac{{dI_{{h_{L} }} }}{dt} = \psi_{h} E_{{h_{L} }} - \left( {\delta_{h} + \mu } \right)I_{{h_{L} }} $$

$$ \frac{{dR_{h} }}{dt} = \delta_{h} (I_{{h_{I} }} + I_{{h_{L} }} ) - \mu R_{h} $$

1 $$ \frac{{dA_{u} }}{dt} = \left[ {\frac{{\rho_{u} F_{u}^{2} + \rho_{w} \left[ {\left( {1 - \gamma } \right)F_{w}^{2} + \left( {1 - \phi } \right)F_{w} F_{u} } \right]}}{F}} \right]\left( {1 - \frac{A}{K}} \right) - \left( {\tau_{u} + \mu_{uA} } \right)A_{u} $$

$$ \frac{{dS_{u} }}{dt} = \frac{{\tau_{u} }}{2}A_{u} - \left( {\beta_{hu} + \mu_{u} } \right)S_{u} $$

$$ \frac{{dE_{u} }}{dt} = \beta_{hu} S_{u} - \left( {\psi_{u} + \mu_{u} } \right)E_{u} $$

$$ \frac{{dI_{u} }}{dt} = \psi_{u} E_{u} - \mu_{u} I_{u} $$

$$ \frac{{dA_{w} }}{dt} = \left[ {\frac{{\rho_{w} \left[ {\gamma F_{w}^{2} + \phi F_{w} F_{u} } \right]}}{F}} \right]\left( {1 - \frac{A}{K}} \right) - \left( {\tau_{w} + \mu_{wA} } \right)A_{w} + \kappa \left( t \right) $$

$$ \frac{{dS_{w} }}{dt} = \frac{{\tau_{w} }}{2}A_{w} - \left( {\beta_{hw} + \mu_{w} } \right)S_{w} $$

$$ \frac{{dE_{w} }}{dt} = \beta_{hw} S_{w} - \left( {\psi_{w} + \mu_{w} } \right)E_{w} $$

$$ \frac{{dI_{w} }}{dt} = \psi_{w} E_{w} - \mu_{w} I_{w} $$

where we have that $F = F_{u} + F_{w}$ is the total adult female mosquito population, and $A = A_{u} + A_{w}$ is the total number of aquatic juveniles.

For the total human population, we have.

$$N_{h} = S_{h} + E_{{h_{I} }} + E_{{h_{L} }} + I_{{h_{I} }} + I_{{h_{L} }} + R_{h}$$ **.**

The total uninfected and *Wolbachia*-infected (adult and juvenile) mosquito populations ($N_{u}$ and $N_{w}$ respectively) are defined as.

$$N_{u} = A_{u} + F_{u} $$ and $$N_{w} = A_{w} + F_{w}$$.

The dengue transmission rates are defined as the multiplication of two parameters: the mosquito biting rate $b_{u\left( w \right)}$; and the dengue transmission probability per mosquito bite $\alpha_{i}$. We then have

$$ \beta_{uh} = \frac{{b_{u} \alpha_{u} I_{u} }}{{N_{h} }} , \beta_{wh} = \frac{{b_{w} \alpha_{wh} I_{w} }}{{N_{h} }}, \beta_{hu} = \frac{{b_{u} \alpha_{u} I_{h} }}{{N_{h} }}, \beta_{hw} = \frac{{b_{w} \alpha_{w} I_{h} }}{{N_{h} }}, $$

where

$$ I_{h} = I_{{h_{I} }} + I_{{h_{L} }} . $$

There are four dengue transmission probabilities per mosquito bite with respect to the model formulation. They are.

- Transmission probability per bite from dengue-infected humans to dengue-susceptible non-*Wolbachia* mosquitoes $\left( {\alpha_{u} } \right)$;
- Transmission probability per bite from dengue-infected humans to dengue-susceptible *Wolbachia*-infected mosquitoes $\left( {\alpha_{w} } \right)$;
- Transmission probability per bite from dengue-infected non-*Wolbachia* mosquitoes to dengue-susceptible humans $\left( {\alpha_{u} } \right)$; and
- Transmission probability per bite from dengue-infected *Wolbachia*-infected mosquitoes to dengue-susceptible humans $\left( {\alpha_{wh} } \right)$.

The transmission probabilities per mosquito bite (a), (b) and (c) are assumed to be the same (i.e., $\alpha_{u} = \alpha_{w}$), however transmission probability (d) is different to the others as *Wolbachia* inhibits dengue virus replication in mosquitoes thereby mitigating transmission.

The pathogen development rates (i.e., the rate at which dengue-exposed *Wolbachia*-infected ($\psi_{w}$) and uninfected ($\psi_{u}$) mosquitoes become actively infectious) and maturation rates for both *Wolbachia*-infected ($\tau_{w}$) and uninfected ($\tau_{u}$) mosquitoes are assumed to be the same, i.e., $\psi_{w} = \psi_{u}$ and $\tau_{w} = \tau_{u}$. Further, the cytoplasmic incompatibility (CI), which describes the mating pair of uninfected female and *Wolbachia*-infected male mosquitoes inability to produce viable offspring and the imperfect maternal transmission of *Wolbachia* infection from *Wolbachia*-infected female mosquito to offspring as described in^36,37^ were also incorporated in the model.

The model (1) is parameterized for Townsville dengue data, however, it can be used for other dengue endemic regions where local dengue outbreaks have occurred as a result of importation of cases. To capture the daily dengue infections generated and their period of occurrence, we defined the parameter $\xi$, as the time-varying dengue monthly importations from 1st January 2001 to 1st February 2019. In addition, these imported dengue cases are assumed to be exposed and not yet infectious. For *Wolbachia* introductions, we defined $\kappa$, as the daily rate that *Wolbachia*-infected mosquitoes are introduced. Therefore, we have that.

$$\kappa \left( t \right) = \left\{ {\begin{array}{*{20}l} {0,} \hfill & {t < T} \hfill \\ {4694,} \hfill & {T \le t \le T^{*} } \hfill \\ \end{array} } \right.,$$

where $T$ is the start time of the *Wolbachia* rollout program in Townsville i.e., $T =$ 1st October, 2014. The *Wolbachia*-infected mosquito releases continued for 28 months and ended on $T^{*} =$ 1st February 2017 (that is, the end time of *Wolbachia*-infected mosquito releases). Remaining model parameters are described in Table 1.

**Table 1:** Model parameters.

Parameter	Description	Value	Dimension	References
$b_{u}$	Biting rate of non-*Wolbachia* mosquitoes	0.3	Per day	^45^
$b_{w}$	Biting rate of *Wolbachia*-infected mosquitoes	0.95 $b_{u}$	Per day	^34^
$\psi_{h}$	Progression rate from exposed to infectious human	1/5.5	Per day	^34^
$\psi_{u\left( w \right)}$	Progression from exposed to infectious non-*Wolbachia* (*Wolbachia*) mosquitoes	0.1	Per day	^34,46^
$\mu$	Human death rate	0.000034	Per day	^45^
$\mu_{uA}$	Death rate of aquatic non-*Wolbachia mosquitoes*	0.02	Per day	^47^
$\mu_{wA}$	Death rate of aquatic *Wolbachia mosquitoes*	0.02	Per day	^47^
$\mu_{u}$	Death rate of non-*Wolbachia* adult mosquitoes	0.043	Per day	^21,37^
$\mu_{w}$	Death rate of *Wolbachia*-carrying adult mosquitoes	0.068	Per day	^21,37^
$N_{h}$	Total human population	187,500	Humans	^35,48^
$L$	Ratio of the mosquito carrying capacity to the total human population	10	–	Assumed
$K_{max}$	Maximum carrying capacity	$2 N_{h}$	Aquatic juveniles	Assumed
$\rho_{u}$	Reproductive rate of non-*Wolbachia* mosquitoes	13	JUVENILES per day	^37,49^
$\rho_{w}$	Reproductive rate of *Wolbachia*-infected mosquitoes	10	Juveniles per day	^37,49^
$\delta_{h}$	Recovery rate	0.2	Per day	^34^
$\alpha_{u}$	Transmission probability per mosquito bite between humans and non-*Wolbachia* mosquitoes	0.1976	–	Estimated
$\alpha_{w}$	Transmission probability per mosquito bite from human to *Wolbachia*-carrying mosquitoes	$\alpha_{u}$	–	Estimated
$\alpha_{wh}$	Transmission probability per mosquito bite from *Wolbachia*-carrying mosquitoes to human	0.0084	–	Estimated
$\tau_{u\left( w \right)}$	Maturation rate of non-*Wolbachia* (*Wolbachia*) mosquitoes	0.11	Per day	^20,50^
$\gamma$	The proportion of *Wolbachia* infected aquatic juveniles resulting from mating between *Wolbachia*-infected mosquitoes	0.95	–	^36^
$\phi$	The proportion of *Wolbachia* infected aquatic juveniles resulting from mating between *Wolbachia*-infected female and non-*Wolbachia* male mosquitoes	0.95	–	^20,37^

#### Seasonal forcing

Here, we adjusted the mosquito carrying capacity (Eq. 2) to account for the seasonal variations in the model as mosquito population fluctuates with climate^42^.

The seasonal varying carrying capacity ($K$) is given as.

2 $$ K = \frac{{LN_{h} }}{2}\left( {\cos \left( {\frac{{2\pi \left( {t - t_{0} } \right)}}{365.25}} \right) + 1} \right) $$

where $L$ is the ratio of the maximum mosquito carrying capacity ($K_{max}$) to the total human population ($N_{h}$), defined by $L = \frac{{K_{max} }}{{N_{h} }}$. The phase shift $t_{0}$ was fixed to match the model simulation to the study location’s seasonal fluctuations.

#### Model simulation procedure with dengue introduction

The model simulations were carried out in R using the general solver for ordinary differential equations “ode” that comes in the “deSolve” package^43^. The initial total population was given as $$N_{h} = S_{h} = 187,500$$ (Townsville population) and other populations are initially set to zero $$ (E_{{h_{I} }} = E_{{h_{L} }} = I_{{h_{I} }} = { }I_{{h_{L} }} = R_{h} = 0)$$. For the initial vector populations, we have that the total adult and aquatic juvenile mosquitoes are $$F = F_{u} + F_{w}$$ and $$A = A_{u} + A_{w}$$ respectively where $$A_{w} + F_{w} = 0 $$ (no *Wolbachia*-positive mosquito introductions yet). We ran the model simulation in four phases.

First, the simulation was run from the first day in 1980 to the last day in the year 2000 with a constant monthly dengue importation rate (computed from the average monthly importations of dengue cases prior to *Wolbachia*-infected mosquito releases) and multiplied by a factor of 4 to account for the inclusion of both symptomatic and asymptomatic dengue cases (1.6 cases/month). This allowed the system to achieve equilibrium in terms of the dengue incidence prior to the simulation period of interest (i.e., 2001 onwards).

Second, we allowed time-varying dengue monthly importations of individuals (dengue events for imported cases) from the first day in January 2001 until February 2019. In other words, rather than the original steady monthly dengue importation, susceptible persons are now exposed to dengue through the monthly-varying importation of dengue-exposed individuals.

Third, for *Wolbachia* introduction, we first initialized the *Wolbachia*-infected mosquito compartments to zero (i.e., $A_{w} = S_{w} = E_{w} = I_{w} = 0$), from the first day of simulation i.e., in the year 1980, as there was no *Wolbachia* introduction at this time ($\kappa = 0$) until 1st October 2014. In what follows, *Wolbachia*-infected mosquitoes’ releases ensued (with $\kappa = 4694$ mosquito introductions per day) and continued for 28 months after which the releases were halted (in February 2017). This led to the interaction between dengue infected humans and mosquitoes in the presence of *Wolbachia* infection with the transmission probabilities per mosquito bite described in Table 1.

Fourth, we fitted the model by comparing the predicted number of symptomatic cases (which we assumed was a quarter of all dengue infections) over the 18-year period (January 2001–February 2019) to the locally acquired dengue case notifications in Townsville. To achieve this, we used a Poisson observation model with mean rate equal to the predicted number of new symptomatic infections per month. We then chose the transmission probabilities per mosquito bite for uninfected and *Wolbachia*-infected mosquitoes $\alpha_{u}$ and $\alpha_{wh}$, as the free parameters and used maximum likelihood (“Nelder-Mead” method in R’s “optim” package) to estimate their central values and generate confidence intervals.

Further, we computed the time-varying reproductive number $R\left( t \right)$, which is the number of new dengue cases generated by a typical infected person in a completely susceptible human population over time by applying the next generation method^44^.

#### Wolbachia intervention efficacy

To compute the overall effectiveness of the *Wolbachia*-infected mosquito rollout, we calculated the percentage reduction in local dengue incidence (in the presence and absence of *Wolbachia*-infected mosquito releases) from the model-predicted values such that we used the model estimates for the dengue transmission probabilities per bite and run a counterfactual scenario in which no *Wolbachia*-infected mosquitoes were introduced. The percentage reduction in the local dengue cases via *Wolbachia* intervention ($\varphi$) was computed using Eq. (3) below:

3 $$ \varphi = \left[ {\frac{{C_{u} - C_{w} }}{{C_{u} }}} \right] \times 100\% , $$

where $C_{u}$ and $C_{w}$ represent the model cumulative dengue incidence in the absence $\left( u \right)$ and presence $\left( w \right)$ of *Wolbachia* mosquitoes respectively from the time *Wolbachia* was introduced until the simulation end-date (February 2019).

## Results

### *Wolbachia* analysis

**Figure 2:**
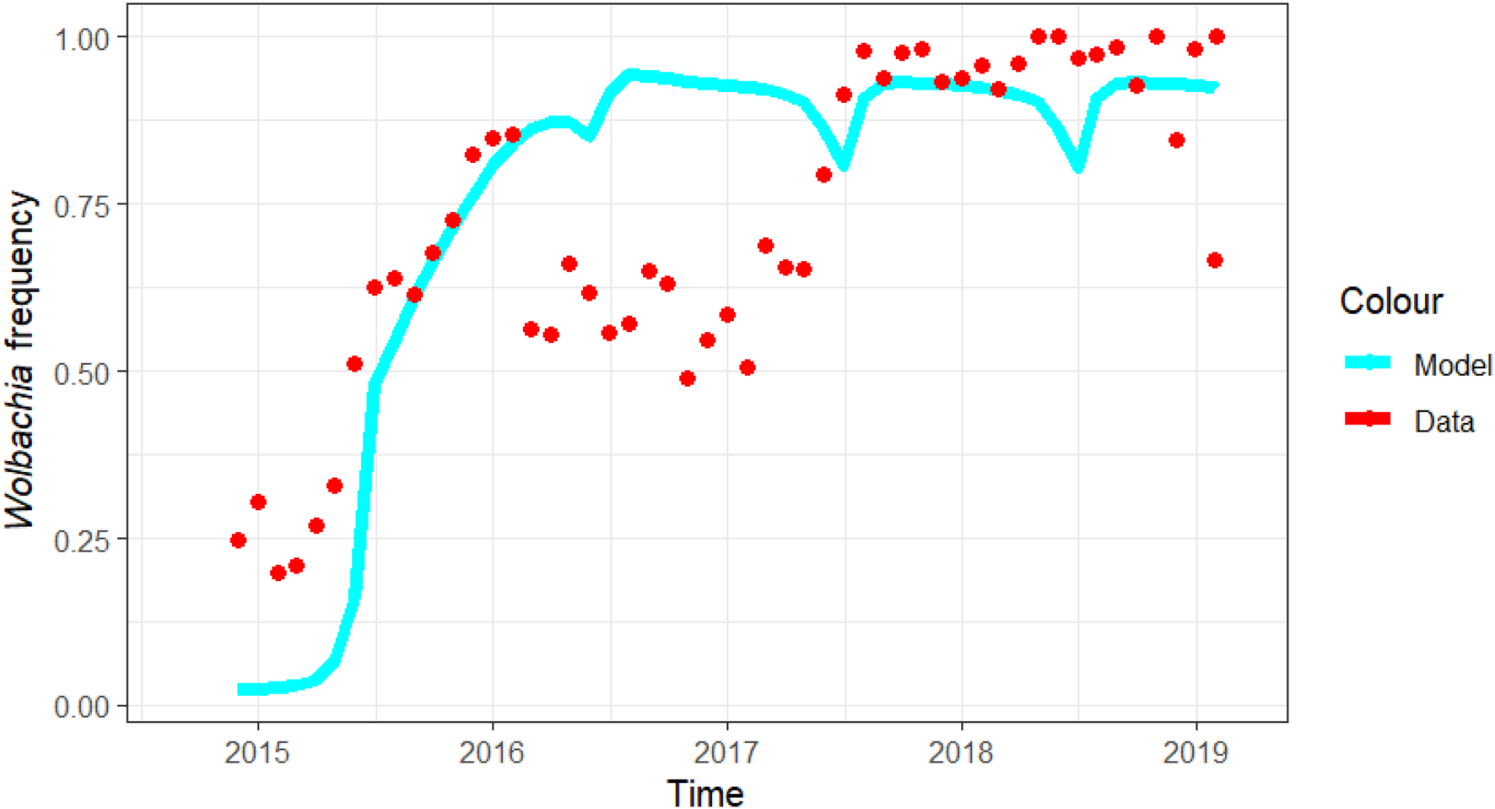
Plot of the proportion of monthly *Wolbachia*-positive mosquitoes along the model simulation to achieve the required *Wolbachia* frequency.

The Townsville *Wolbachia* data, which includes the release period, mosquitoes’ collection date, number of mosquitoes collected, proportion of *Wolbachia* positive mosquitoes and the rollout location (suburbs)^14^ were compiled into monthly data to show the monthly distribution of *Wolbachia*-infected mosquitoes from the total captured mosquitoes from October 2014 to February 2019 (Fig. 2). It is observed that there was an increasing trend in the *Wolbachia* positive mosquitoes indicating increased establishment of *Wolbachia*-infected mosquitoes.

In this *Wolbachia* analysis, no fitting was performed: the results emerged from the baseline parameter values obtained from existing literature. By leveraging the wealth of established knowledge already available, we were able to establish a solid foundation for our analysis without the need for additional adjustments or tuning. The fluctuations in the model-predicted *Wolbachia*-frequency arose from both the competitive dynamics of the wild-type and *Wolbachia*-infected mosquito populations, and our choice to model the mosquito carrying capacity $K$ as a sinusoidal function of time (Fig. 2). For reference, the individual numbers of *Wolbachia*-infected and uninfected mosquitoes and the corresponding *Wolbachia* frequency are captured in Figures A1, A2 and A3 of the Supplementary file respectively.

### Basic reproductive number (${\varvec{R}}_{0}$)

The reproductive number in the presence of *Wolbachia*-infected mosquitoes ($R\left( t \right)$) is given as:

$$ R\left( t \right){ } = \sqrt {R_{u} \left( t \right)^{2} + R_{w} \left( t \right)^{2} } , $$

where $R_{u} \left( t \right)$ and $R_{w} \left( t \right)$ are the dengue reproductive numbers of the uninfected and *Wolbachia*-infected mosquito populations (see Supplementary file, Appendix A1), given respectively as

$$ R_{u} \left( t \right) = \sqrt {\frac{{b_{u}^{2} \alpha_{u}^{2} \psi_{u} \psi_{h} S_{u} \left( t \right)}}{{\left( {\mu_{u} + \psi_{u} } \right) (\mu + \delta_{h} ) \left( {\mu + \psi_{h} } \right)\mu_{u} N_{H} }}} $$

and

$$R_{w} \left( t \right) = \sqrt {\frac{{b_{w}^{2} \alpha_{wh} \alpha_{u} \psi_{w} \psi_{h} S_{w} \left( t \right)}}{{\left( {\mu_{w} + \psi_{w} } \right) (\mu + \delta_{h} ) \left( {\mu + \psi_{h} } \right) \mu_{w} N_{H} }}}.$$

In the absence of *Wolbachia*-infected mosquitoes, $R\left( t \right){ } = R_{u} \left( t \right)$.

### Parameter estimation

**Figure 3:**
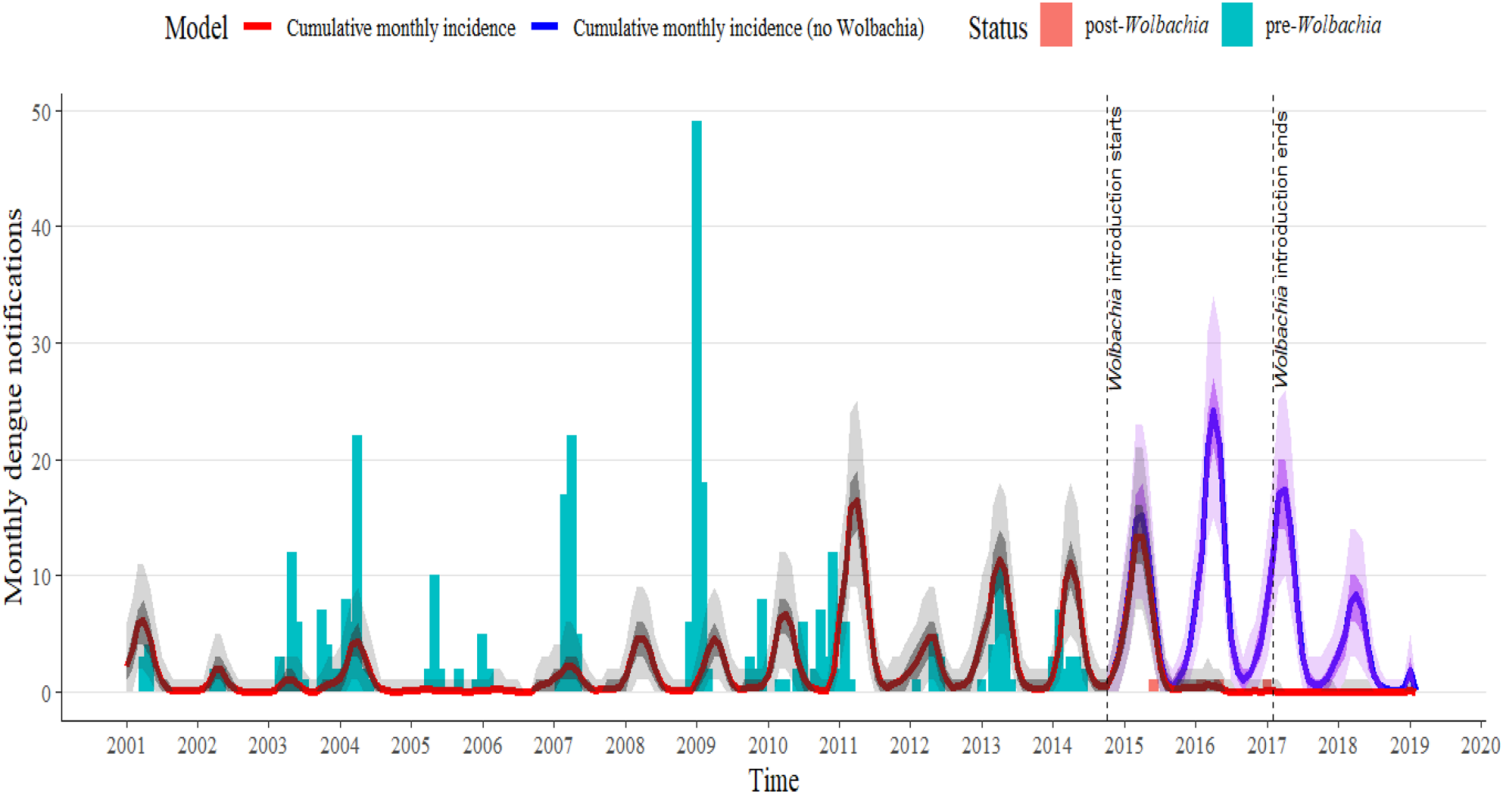
Townsville locally acquired dengue cases’ data in the presence (red bars) and absence (green bars) of *Wolbachia* releases from 2001 to 2019 together with the predicted dengue incidence in the presence (red line) and absence (blue line) of *Wolbachia*-infected mosquitoes using the model (1) with 50% (dark grey/purple) and 95% (light grey/purple) confidence intervals for the parameter uncertainty. From the start to end date of the *Wolbachia*-infected mosquito introduction (the period between the first and second black dashed vertical lines), the monthly dengue incidence decreased by 65% and after the *Wolbachia* intervention (from the second black dashed line to the right), the dengue incidence had reduced by 99% in comparison to the counter-factual scenario in which *Wolbachia*-infected mosquitoes are not introduced.

In the model (1), we have fitted the predicted monthly cumulative dengue incidence from January 2001 to February 2019 to the locally acquired dengue case notifications in Townsville. The maximum likelihood estimates for the transmission probability per bite from dengue-infected *Wolbachia*-uninfected mosquitoes to susceptible humans is $\alpha_{u}$ = 0.1976 (CI 0.1966–0.1986) and from dengue-infected *Wolbachia*-infected mosquitoes to susceptible humans is $\alpha_{wh}$ = 0.0084 (CI 0.0079–0.0090) (Fig. 3). The estimate $\alpha_{u}$ is consistent with the modelling study estimate in^34^ carried out in Cairns.

Figure 3 shows that dengue infections occur mostly in the summer and usually die out in the winter following the seasonal forcing of the model. Further, in the data, there is an increase in local cases from 2007 to 2011 with the highest number recorded in early 2009. However, when *Wolbachia* was introduced in the last quarter of 2014, there was a drastic reduction in local dengue cases. The corresponding reduction in dengue cases via *Wolbachia* intervention ($\varphi$), which was computed using Eq. (3) within the *Wolbachia*-infected mosquitoes’ release period (between October 2014 and February 2017) is 65.47% (CI 65.17–65.70%). After the *Wolbachia*-infected mosquito releases were halted in February 2017, the observed dengue cases had reduced by 99.32% (CI 99.26–99.40) compared to the counterfactual scenario in which *Wolbachia* was not introduced. Figure 3 describes the model fits in the presence and the counter-factual (absence) of *Wolbachia*-infected mosquitoes. In the counter-factual scenario, the increased predicted cases follow from an increase in the number of imported cases. The separate model fits in the presence and absence of *Wolbachia*-infected mosquitoes can be found in Figures A4 and A5 respectively in the Supplementary file.

**Figure 4:**
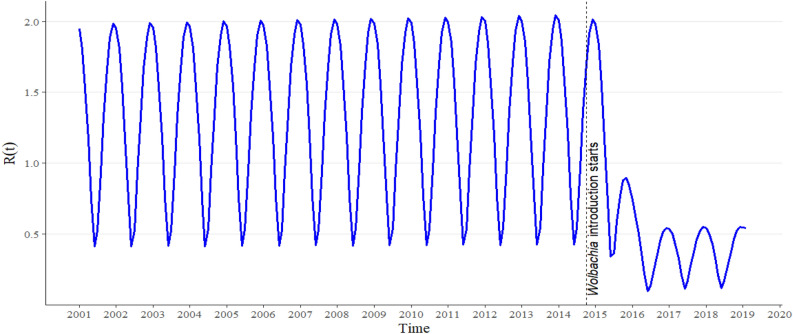
Time-varying reproductive number ($R\left( t \right)$).

To account for the impact of *Wolbachia* introduction on dengue infection, we further computed the reproductive number ($R\left( t \right)$) in the presence of *Wolbachia* mosquitoes using the estimated transmission probabilities per mosquito bite (Fig. 4).

Prior to *Wolbachia* introduction in Fig. 4, the peak $R\left( t \right)$ is ~ 2.04. This is consistent with the reproductive number estimated in Cairns, North Queensland in 2008^51^. This indicates that dengue infection keeps circulating through the years as a result of continual influx of dengue imported cases. Further, after *Wolbachia* introduction in October 2014, within 2 years, there was a drastic decrease in the peak $R\left( t \right)$, which becomes ~ 0.55. In practice, the dengue infection should gradually die out as shown in Fig. 4, bringing the number of cases to nearly zero, however, it didn’t as a result of continual import of dengue infected persons.

**Figure 5:**
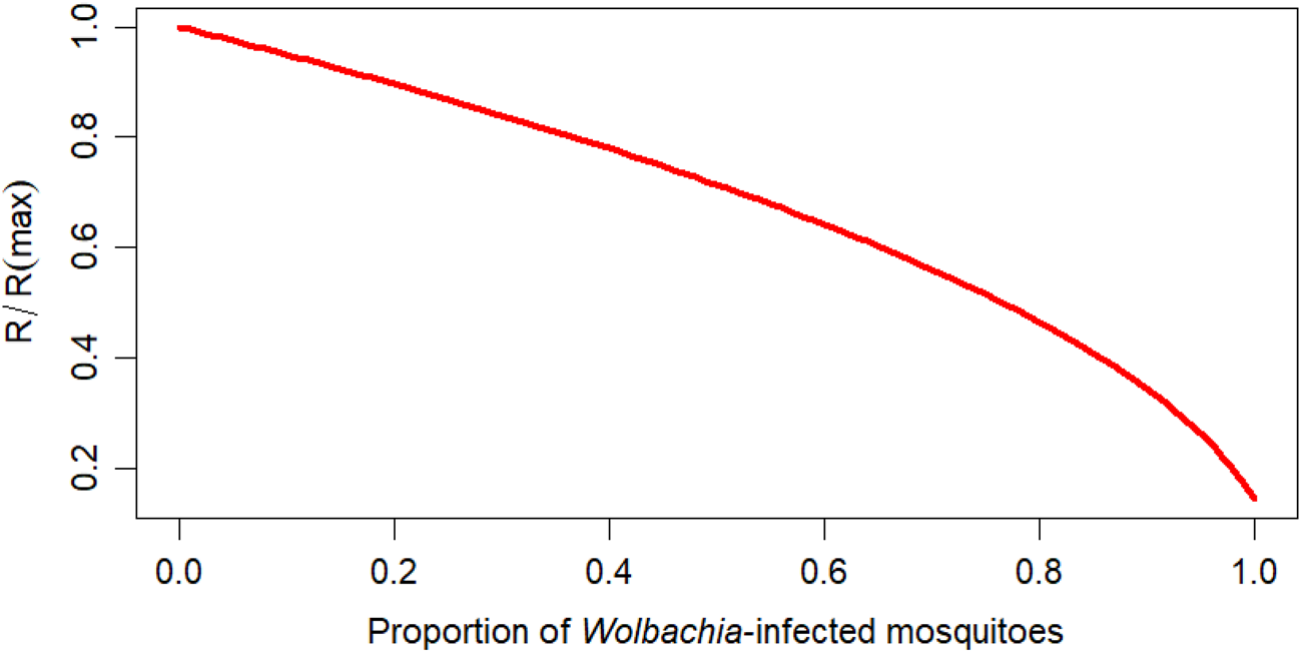
Shows the relationship between the relative reproductive number in the presence of *Wolbachia-*mosquitoes and the proportion of *Wolbachia*-infected mosquitoes.

Using our estimates for the dengue transmission probabilities per bite from wild-type and *Wolbachia*-infected mosquitoes, we calculated the relative reduction in the reproduction number as a function of the frequency of adult *Wolbachia* mosquitoes in the vector population (see Appendix A2 in Supplementary file). The results are shown in Fig. 5, from which we can see an accelerating downward trend in the relative reproductive rate $R\left( \eta \right)$, with increasing *Wolbachia* frequency ($\eta$), reaching a minimum of near 0 (0.143) if all the wild-type mosquitoes are replaced with *Wolbachia*-infected mosquitoes. Note, that in producing Fig. 5 we have made the simplifying assumption that in the transition from a purely wild-type mosquito population to one dominated by *Wolbachia*-infected mosquitoes, the total vector population remains constant. In reality, the vector population slightly decreases across this transition (as a result of the increased death rate of *Wolbachia*-infected mosquitoes), meaning that the red-curve in Fig. 5 should be treated more as an upper bound on the relative reproductive number.

Further, our simulation showed that the *Wolbachia* frequency (that is, the percentage of wild-type mosquitoes being replaced by the *Wolbachia*-infected mosquitoes) had increased to over 90% at the end of halting the *Wolbachia* releases in February 2017, and the frequency was maintained until February 2019. This corresponds to the observation that on introducing *Wolbachia*-infected mosquitoes ($S_{w}$) from October 2014 to the end of the study in February 2019, the peak $R$ was reduced by 73% (Fig. 5). In the case of high $R$, introducing *Wolbachia*-infected mosquitoes will decrease the dengue incidence but may still not reduce $R$ below 1^34,52^. This showed that *Wolbachia* rollout works but may not fully eradicate dengue infections in some high dengue endemic settings depending on *Wolbachia's* ability to sufficiently reduce $R$ less than one as verified experimentally by^17^.

### Sensitivity analysis

**Figure 6:**
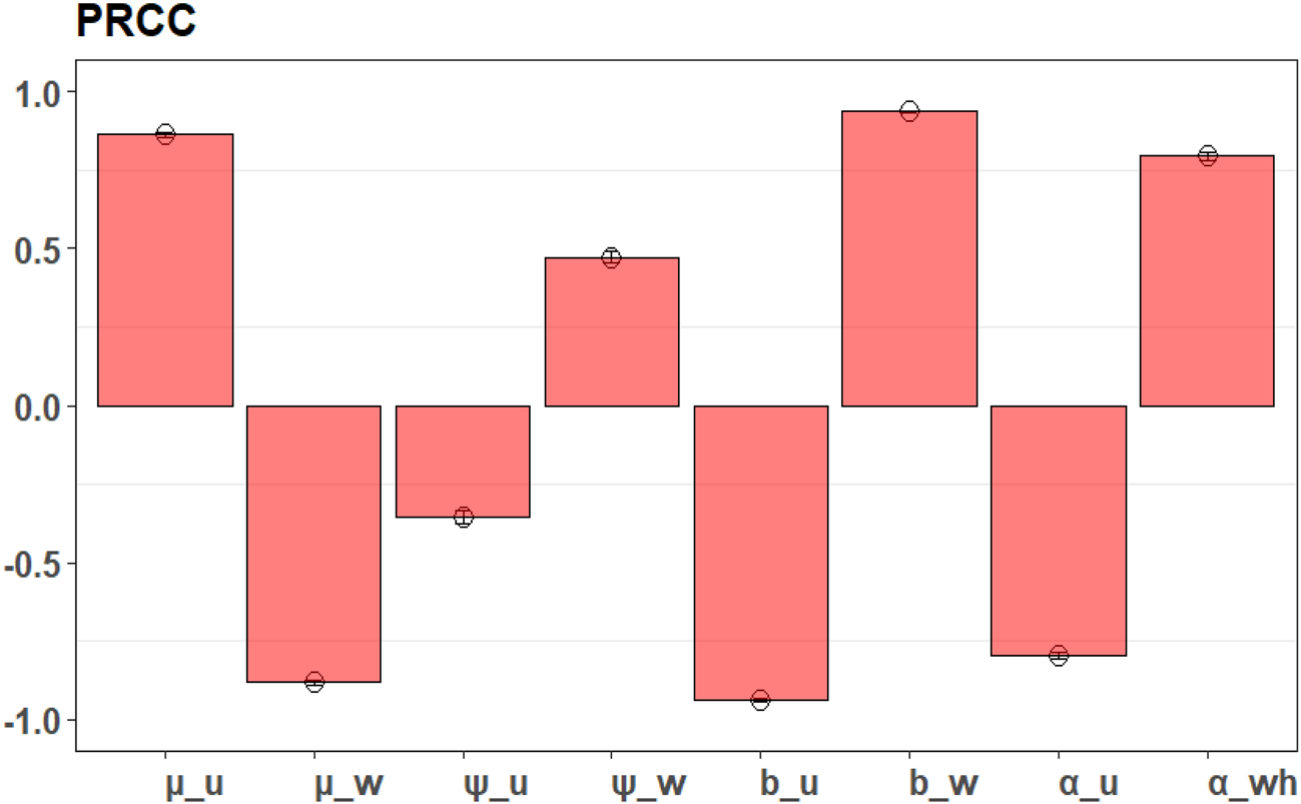
PRCC values illustrating the sensitivity of the model output, $R\left( \eta \right)$—the relative reproductive number as a function of *Wolbachia*-infected mosquitoes’ proportion for the estimated parameters $\mu_{u} ,\mu_{w} ,\psi_{u} ,\psi_{w} ,b_{u} ,b_{w} ,\alpha_{u} ,\alpha_{wh} .$

In this section, we perform a sensitivity analysis to determine which parameters have the most influence on outputs of the model. Here, we use the partial rank correlation coefficient (PRCC) estimation^53^, an effective and efficient sampling-based approach to compute our estimates via the “pcc” function in the “sensitivity” package in R. The parameters are sampled at random with 10,000 simulations, under a uniform distribution and a 30% deviation from the baseline values. Simulation results are illustrated in Fig. 6. According to the positive or negative correlation, changing the parameter in a way that is either positive or negative will result in an improved or worsened model result respectively. In this study, the relative reproductive number, which is a function of the proportion of *Wolbachia-*infected mosquitoes $R\left( \eta \right)$ is the model output we take into consideration with a 90% proportion of *Wolbachia*-infected mosquitoes. Figure 6 shows the PRCC estimates of $R\left( \eta \right)$ which corresponds to the parameters $\mu_{u} ,\mu_{w} ,\psi_{u} ,\psi_{w} ,b_{u} ,b_{w} ,\alpha_{u} ,\alpha_{wh}$ in our model. In Fig. 6, the parameters $\mu_{u} ,\psi_{w} ,b_{w} ,\alpha_{wh}$ have positive and $\mu_{w} ,\psi_{u} ,b_{u} ,\alpha_{u}$ negative PRCC values. This suggests that an increase in these positive parameters will result in an improvement in the $R\left( \eta \right)$, while an increase in the negative parameters would worsen the $R\left( \eta \right)$.

## Discussion

In this study, we developed a mathematical model of dengue infection dynamics in humans together with mosquito population dynamics in the presence of *Wolbachia* infection and investigated the pre- and post-*Wolbachia* effects on dengue-infected individuals. We observed that for the imported dengue case data, the imported cases were significantly lower during the pre-*Wolbachia* times (from January 2001 to September 2014) than post-*Wolbachia* times (October 2014–February 2019). This likely reflects global trends in which there was a resurgence of dengue cases throughout the South-East Asia region (from 2015 to 2019)—the source location of most international importations of dengue into Townsville^1,54,55^. Despite higher numbers of dengue introductions, local dengue dropped significantly after *Wolbachia* introduction.

Further, the results of the parameter estimation showed the transmission probability per bite from *Wolbachia* mosquitoes to susceptible humans is reduced by a factor of approximately 20, relative to that of non-*Wolbachia* infected mosquitoes. With the estimated transmission rates, *Wolbachia* was able to reduce dengue incidence during the *Wolbachia*-infected mosquito releases i.e., between October 2014 and February 2017 (28 months) by 65.47% (CI 65.17–65.70%) and after the *Wolbachia* intervention from February 2017 to February 2019 (24 months) by 99.32% (CI 99.26–99.40%).

There are some limitations to this study. First, in our model, we did include seasonality in the mosquito carrying capacity but not temperature changes which could influence the loss of *Wolbachia* infection. Although including seasonal changes, which could be driven by both rainfall and temperature changes as rainfall may have a varying effect on the mosquito abundance, neglecting temperature in this study maintains *Wolbachia* infections in mosquitoes. Second, we have only considered a single circulating serotype of dengue in humans and mosquito vectors in the presence of a *Wolbachia* endosymbiont. Further studies may consider factors such as co-circulation of different serotypes of dengue virus in the presence of different *Wolbachia* strains and investigate the impact on the dengue transmission dynamics. Finally, we have made some assumptions based on published research about the parameters employed in this work. This may affect the reproductive number.

Additionally, in most climes, if the reproductive number is less than one, it means that the infection rate will eventually fall to zero. However, this may not always hold as several factors may contribute to this effect in the class of models such as ours. These factors include model stochasticity that may be sensitive to random events and backward bifurcation, and as such, the reproductive number being less than one does not ensure a complete absence of dengue disease, as repeated importations may continue to cause stuttering chains of transmission.

Our model also examined the sensitivity of parameters for a 90% *Wolbachia*-infected mosquitoes’ proportion to the model output such as the relative reproductive number. It is evident that the model output is dependent on the $\mu_{u} ,\mu_{w} ,\psi_{u} ,\psi_{w} ,b_{u} ,b_{w} ,\alpha_{u} ,\alpha_{wh}$ parameters. Of the parameters, $\mu_{w}$ and $b_{w}$ are the most sensitive. To control and eradicate dengue, we need to consider the following strategies: by minimizing the biting rate, the transmission probabilities per bite and the pathogen development rates of the *Wolbachia*-infected mosquitoes.

Although our model does not appear to produce the predicted good fit as expected, there are several underlying factors that may contribute to this. These factors include environmental factors such as temperature variations, humidity, and rainfall which were not incorporated in the model, as these may have influenced the survival and propagation of *Wolbachia*-infected mosquitoes and the dengue virus they transmit. As such, the proportion of *Wolbachia*-positive mosquitoes may not correlate well with the *Wolbachia* frequency in the model, and this may influence disease incidence. Other factors that may influence the model fit include the human interventions such as introducing other vector control measures e.g., the use of insecticides or insecticide-treated bed nets or changes in human behaviour. Continuous research and monitoring are required to better understand the intricate relationships between *Wolbachia* bacteria, *Aedes* mosquitos, arboviruses-dengue virus, and the environment in order to solve these difficulties. Such knowledge can be used to develop ways to increase the efficacy of *Wolbachia*-based therapies and improve fit for the proportion of *Wolbachia* frequency and dengue disease incidence.

The findings from this study have demonstrated consistency with the study in Cairns^34^ in terms of the impact of *Wolbachia* introductions in reducing dengue cases. Our results showed that *Wolbachia* intervention may be successful in reducing dengue outbreaks if the reproductive number $R\left( t \right)$ is less than one after intervention. We also showed that *Wolbachia*-mosquito introduction may successfully replace the wild-type mosquitoes depending on *Wolbachia's* ability to sufficiently reduce $R\left( t \right)$ less than one.

In addition, we have found that the impact of *Wolbachia* rollout has been durable across the study period, but we also showed that pre-*Wolbachia*, peak annual reproductive number $R$ is ~ 2 and to maintain this less than one for the whole year we would need to maintain *Wolbachia*-infected mosquitoes at 75%.

In conclusion, the results of this work showed that *Wolbachia* release can be successful in reducing the incidence of dengue in areas with low or moderate endemicity provided that there is a low chance of dengue transmission from *Wolbachia*-infected mosquitoes (that is, transmission probabilities per bite: 0.0079–0.0090) together with biologically realistic parameters as described in Table 1. This work will contribute to the understanding of dengue transmission rates as part of the global effort to dramatically mitigate dengue transmission.

### Supplementary Information

Supplementary Information.

## Data Availability

The datasets generated during and/or analysed during the current study are available in the following published paper^14^, [https://dx.doi.org/10.6084/m9.figshare.8282306.v1]. The open source code used for this project is publicly available at https://github.com/samsontosin/quantifying_the_impact.
