# Supplementary material for: Quantifying the impact of Wolbachia releases on dengue infection in Townsville, Australia
Source: Sci Rep. 2023 Sep 11;13:14932. doi: 10.1038/s41598-023-42336-2 (PMC10495365; doi:10.1038/s41598-023-42336-2)
Supplement: Supplementary file 1 — Supplementary Information. [file 41598_2023_42336_MOESM1_ESM.docx]

**Appendix**

**A1.**

Here, we derive the basic reproductive number $R(t)$ – the number of new dengue cases that an infected individual would cause in a community of humans that are entirely susceptible, by using the next generation method and calculating its spectral radius [1]. At the dengue infection-free steady state, $E_{h_{I}}=E_{h_{L}}=I_{h_{I}}=I_{h_{L}}=R_{h}=0, E_{u}=I_{u}=0, E_{w}=I_{w}=0.$ Therefore, $N_{h}=S_{h}$. To compute $R(t)$ with or without *Wolbachia-*infected mosquitoes, we divide the Exposed and Infected local humans and vector compartments into the appearance rate of new dengue infections$, f$, and progression rates from exposed to infectious compartments and death rates, $v$.

Let $F_{ij}=\frac{\partial f_{i}}{\partial x_{j}}$ and $V_{ij}=\frac{\partial v_{i}}{\partial x_{j}}$ where $x_{j}'s$ are the exposed and infected compartments respectively. Therefore,

$F\boldsymbol{=}\left( \begin{matrix} 0 & 0 & 0 & Lb_{u}\alpha_{u} & 0 & {Lb}_{w}\alpha_{wh} \\ 0 & 0 & 0 & 0 & 0 & 0 \\ 0 & b_{u}S_{u}\alpha_{u} & 0 & 0 & 0 & 0 \\ 0 & 0 & 0 & 0 & 0 & 0 \\ 0 & b_{w}S_{w}\alpha_{w} & 0 & 0 & 0 & 0 \\ 0 & 0 & 0 & 0 & 0 & 0 \end{matrix} \right)$ and

$V\boldsymbol{=}\left( \begin{matrix} {\mu+\psi}_{h} & 0 & 0 & 0 & 0 & 0 \\ -\psi_{h} & \mu+\delta_{h} & 0 & 0 & 0 & 0 \\ 0 & 0 & \mu_{u}+\psi_{u} & 0 & 0 & 0 \\ 0 & 0 & -\psi_{u} & \mu_{u} & 0 & 0 \\ 0 & 0 & 0 & 0 & \mu_{w}+\psi_{w} & 0 \\ 0 & 0 & 0 & 0 & -\psi_{w} & \mu_{w} \end{matrix} \right)$.

Hence the reproductive number in the presence of *Wolbachia*-infected mosquitoes ($R_{0}$) is given as:

$R(t) =\sqrt{{R_{u}}^{2}+{R_{w}}^{2}}$, where,

$R_{u}(t)=\sqrt{\frac{b_{u}^{2}\alpha_{u}^{2}\psi_{u}\psi_{h}S_{u}(t)}{\left( \mu_{u}+\psi_{u} \right){{(\mu+\delta}_{h}) (\mu+\psi_{h})\mu}_{u}N_{H}}}$ and $R_{w}(t)=\sqrt{\frac{b_{w}^{2}\alpha_{wh}\alpha_{u}\psi_{w}\psi_{h}S_{w}(t)}{\left( \mu_{w}+\psi_{w} \right){(\mu+\delta}_{h}) (\mu+\psi_{h}) \mu_{w}N_{H}}}$,

while in the absence of *Wolbachia*-infected mosquitoes, $R(t)=R_{u}(t)$.

**A2**

To account for $R(t)$ with respect to the proportion of *Wolbachia*-infected mosquitoes $\eta$, we have that

$$R\left( t \right)=\sqrt{\chi\left( S_{u}\left( t \right)+\sigma S_{w}\left( t \right) \right)}$$

where

$$\chi=\frac{b_{u}^{2}\alpha_{u}^{2}\psi_{u}\psi_{h}}{\left( \mu_{u}+\psi_{u} \right){{(\mu+\delta}_{h}) (\mu+\psi_{h})\mu}_{u}N_{H}}$$

$$\sigma=\frac{\frac{b_{w}^{2}\alpha_{wh}\psi_{w}}{\left( \mu_{w}+\psi_{w} \right) \mu_{w}}}{\frac{b_{u}^{2}\alpha_{u}\psi_{u}}{\left( \mu_{u}+\psi_{u} \right)\mu_{u}}}$$

The independent variable $\eta$, which is the *Wolbachia*-infected mosquitoes’ fraction is given as:

$$\eta=\frac{S_{w}\left( t \right)}{S_{u}\left( t \right)+S_{w}(t)}$$

Assume that $S_{u}+S_{w}=C$ is constant,

$$\eta=\frac{S_{w}}{C}\to S_{w}=\eta C$$

$$1-\eta=\frac{S_{u}}{C}\to S_{u}=\left( 1-\eta\right)C$$

We then have

$$R(\eta)=\sqrt{\chi C\left( \left( 1-\eta\right)+\sigma\eta\right)}$$

$$R\left( 0 \right)=\sqrt{\chi C}$$

$$\frac{R\left( \eta\right)}{R\left( 0 \right)}=\sqrt{\left( 1-\eta\right)+\sigma\eta}$$

If $\eta=1$, that is, if all wild-type mosquitoes are replaced by *Wolbachia*-infected mosquitoes,

$$\frac{R\left( \eta\right)}{R\left( 0 \right)}=\sqrt{\sigma}$$

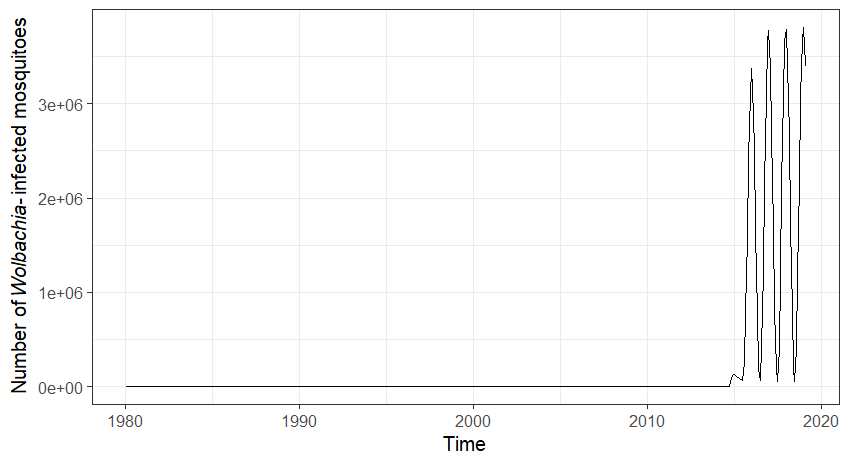


Figure A1: Graph showing the number of *Wolbachia*-infected mosquitoes over time.


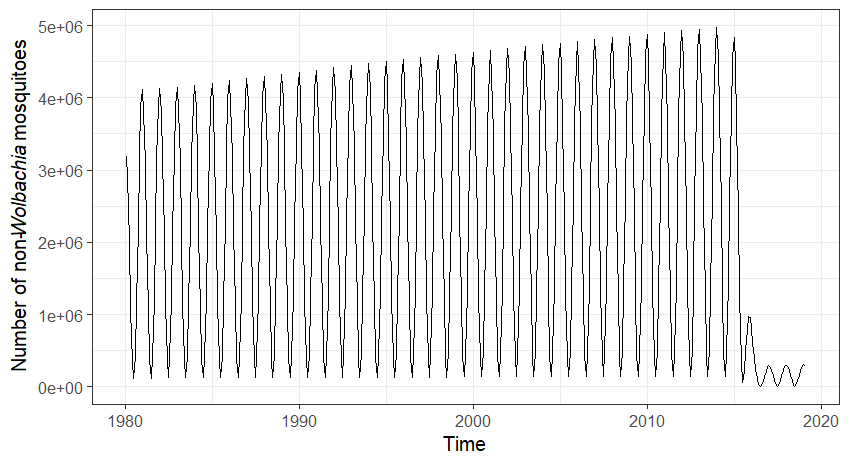


Figure A2: Graph showing the number of uninfected mosquitoes over time.


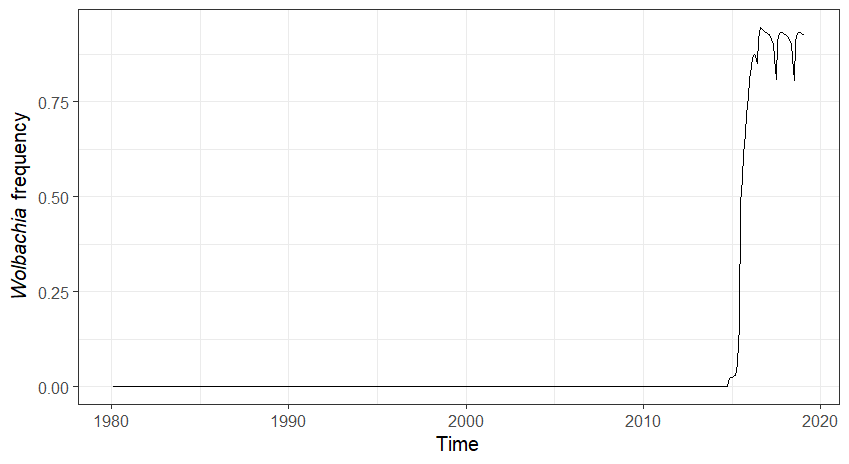


Figure A3: Graph showing the *Wolbachia* frequency over time.


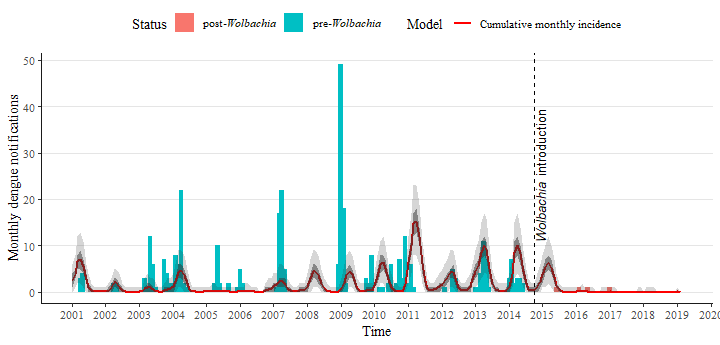


Figure A4: Plot of the data fitting of the number of Townsville locally acquired dengue cases in the presence of *Wolbachia* mosquitoes from 2001-2019 using the model (1) with 50% (dark grey) and 95% (light grey) confidence interval.


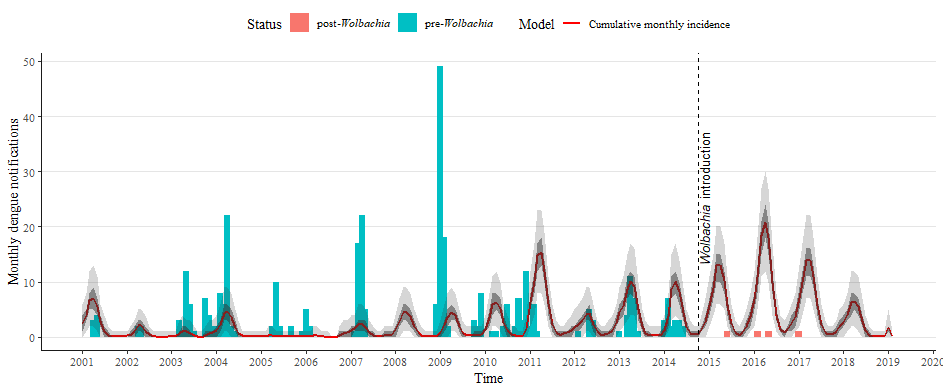


Figure A5: Plot of the data fitting of the number of Townsville locally acquired dengue cases in the absence of *Wolbachia* mosquitoes from 2001-2019 using the model (1) with 50% (dark grey) and 95% (light grey) confidence interval.

1. van den Driessche, P. and J. Watmough, *Reproduction numbers and sub-threshold endemic equilibria for compartmental models of disease transmission.* Math Biosci, 2002. **180**: p. 29-48.
